# Supplementary material for: Are carbon emissions trading and green financial instruments synergistic? -Comprehensive quantitative research based on content analysis
Source: PLoS One. 2024 Mar 7;19(3):e0298601. doi: 10.1371/journal.pone.0298601 (PMC10919601; doi:10.1371/journal.pone.0298601)
Supplement: S1 Data — (ZIP) [file pone.0298601.s001.zip › Data/地级市Z_F工作报告.zip!/░▓╗╒/║╧╖╩╩╨/└┤╫╘╣1⁄2╓┌║┼í╛┬φ┐╦╩2╛▌═°í┐_2009.docx]

# **2009年政府工作报告**

发布时间：2009-01-07 10:06

【字体：[大](http://www.hefei.gov.cn/public/1741/javascript:void(0);) [中](http://www.hefei.gov.cn/public/1741/javascript:void(0);) [小](http://www.hefei.gov.cn/public/1741/javascript:void(0);)】

各位代表：

　　现在，我代表市人民政府，向大会作政府工作报告，请予审议，并请市政协委员和其他列席人员提出意见。

　　一、2008年工作回顾

　　刚刚过去的2008年，是合肥发展史上极不平凡的一年。一年来，我们众志成城，战胜了历史罕见的雨雪冰冻灾害；沉着应对，及时遏止了突如其来的手足口病疫情；万众一心，全力救助和支援汶川地震灾区；精心组织，圆满完成世人关注的奥运火炬传递任务；特别是，面对国际金融危机对经济发展的影响，全市人民在省委、省政府和中共合肥市委的坚强领导下，全面贯彻党的十七大、十七届三中全会和胡锦涛总书记视察安徽重要讲话精神，以科学发展观为指引，认真贯彻落实中央及省各项方针政策，坚定发展信心，创新发展举措，有效应对并化解各种突出矛盾和问题，全面超额完成了市十四届人大一次会议确定的年度主要目标任务，在现代化滨湖大城市建设进程中迈出了坚实步伐。

　　预计，全市地区生产总值1630亿元，比上年增长17%；财政收入301。2亿元，增长40%，其中地方财政收入160。9亿元，增长57。8%；市国税组织收入143。5亿元，增长20。9%，地税组织收入99。1亿元，增长50%；全社会固定资产投资1835亿元，增长40%；社会消费品零售总额587亿元，增长25%；城镇居民人均可支配收入15550元，农民人均纯收入5300元，分别增长15。8%和18%。主要经济指标增速继续位居全国省会城市前列。城镇登记失业率4。1%；人口出生率10。2‰；居民消费价格指数106。5%。单位GDP能耗及主要污染物减排量均完成省控目标。各项事业全面发展，社会大局和谐稳定。总体看，在极其复杂的宏观环境下，全市经济社会仍然保持又好又快的发展势头，厚积薄发、加速崛起已成为新时期合肥发展的重要特征。

　　一年来的主要工作是：

　　(一)以企业为核心、项目为抓手，工业立市累积效应显现

　　坚持把扩大有效投入作为促增长、增后劲的关键举措，千方百计争项目、促投资，一批重大项目加快推进。全年累计向国家和省申报项目339项，总投资872。6亿元。特别是中央扩大内需政策出台后，我们快速反应，上报项目125个。全年争取国债资金8。1亿元，是2007年的3。3倍。市"1346"行动计划项目完成投资635亿元，增长35。7%，其中，列入省"861"行动计划项目完成投资432亿元，增长一倍。

　　以企业为工业发展核心，强化政府支持与服务。进一步完善新型工业化政策体系，全年兑现专项资金6亿元，增长65。8%；免收开发园区、乡镇工业聚集区工业投资项目行政事业性收费3亿元。强化工业发展投入，全年完成工业投资523亿元，增长56%。京东方六代线、熔安动力、大陆轮胎、合肥电厂6#机组等一批超20亿元重大项目前期工作取得突破性进展，美的冰洗工业园、格力产业基地一期、长虹工业园等建成投产。切实加强工业运行调度，及时出台促进经济平稳较快增长的政策措施。每月召开经济运行调度会，不定期对重大项目进行调度；实行项目落地开工周报制和市领导直接联系重大项目制度；在市委统一调度下，派出60个驻企服务工作组，上门帮助企业解决困难和问题；加强与在肥企业总部沟通，全力促进增产增收增效；向金融机构分三批推介优质企业近900户，建立金融超市网，有效缓解了中小企业流动资金需求压力。预计，全年规模以上工业完成总产值2070亿元，实现增加值605亿元，增长25%；八大产业实现产值1430亿元，增长29%；新增规模以上工业企业311户，产值超亿元企业达到275户。

　　强化现代服务业发展支撑作用，出台支持服务业发展的实施意见和五十条具体政策，重点扶持14个领域。加大金融工作力度，金融机构对地方经济支持力度进一步加大，当年新增贷款量占全省43。8%。东亚银行、民生银行来肥设立分支机构。新开工大型商贸重点项目37个，现代物流基地、物流配送中心等加速推进。会展经济蓬勃发展，全年举办广告节、家博会等各类会展120场。特色产品开发及宣传促销成效显著，全年实现旅游总收入130亿元。

　　(二)坚持城乡统筹、加快结构调整，县域经济发展强劲

　　全面贯彻落实十七届三中全会精神，出台推进农村改革发展、加速城乡一体化进程的实施意见和支持现代农业发展的五十三条具体政策，加大对县域经济发展和新农村建设支持力度。全年累计投入涉农资金14。2亿元，其中市级财政预算内资金5。4亿元，增长23%。落实强农惠农政策，民生工程及社会事业向县域倾斜，城市路水电气等基础设施加快向县域延伸，一半以上乡镇开通公交车。

　　县域经济在结构调整中快速发展。三县主要经济指标增速高于全市平均水平，规模以上工业总产值、固定资产投资均突破百亿元，县域经济对全市经济增长的贡献率达24%。县域工业发展势头强劲，食品及农副产品加工业产值突破200亿元，一批乡镇工业园区迅速崛起。现代农业呈现出规模化、特色化发展特征。全市粮食总产量184。3万吨，创历史新高。新增设施栽培小区5500亩、露地菜生产基地2。1万亩、苗木花卉1。9万亩；新增畜禽规模养殖场(区)216个。"一村一品"专业示范村发展到114个；以农家乐为特色的旅游农业点发展到118家。完成外环森林生态长廊一期工程，林业资源总量突破100万亩。新增省级以上龙头企业11家、产值超亿元龙头企业12家、超10亿元2家；新增农民专业合作经济组织139家，对农户带动面达48%；新增农业标准化示范基地20个，新认定无公害农产品20个。

　　新农村建设继续扎实推进。"十镇百村"工程市级示范点扩大到152个。四个"整村推进"土地整理及新农村建设项目成效明显，九个农民集中住宅区初步建成。"村庄环境整治"、"四清四改"、"生态村创建"以及"清洁家园、绿化乡村"千村百日行动深入开展，农村生态及农民居住环境显著改善。农村基础设施建设进一步加强。新建通乡油路175公里，农村公路总里程突破8000公里；村村通水泥路率、通班车率和通自来水率分别达95%、96%和80%；完成55座水库除险加固工程，解决21万人口安全饮水问题。

　　(三)"141"战略加速实施，滨湖大城市建设扎实推进

　　强化规划引领功能，编制省会经济圈城镇体系规划，启动合淮同城化规划，完成37项专项规划。被批准为全国第一个节约集约用地试点市。全年完成大建设投资206。6亿元，新建、续建项目323个，建成道路218。8公里，城市功能进一步完善，城市品位和形象进一步提升。

　　区域综合交通枢纽加快形成。合宁高铁正式运营，合武高铁建成通车，合蚌高铁及铁路南站搬迁建设启动；新桥国际机场、合肥港综合码头、派河码头及航道疏浚工程开工建设；105公里的外环高速全线贯通，"一环六射"高速路网正式形成；合六路等城市出入口道路建成通车，合店路一期工程等开工建设。

　　路桥建设向城市核心区拓展。全年新建、改建道路45条、桥梁48座。长江中路综合改造全面完成，安徽第一路面貌焕然一新；宿州北路、清溪东路、长江东大街、临泉路东段、紫云路等竣工或即将完工，主城区骨干路网由"十字型"转为"井字型"布局；北一环、二环改造工程顺利竣工。城市环境综合整治成效显著。南淝河流域河道全面实施截污，铺设管网560多公里；蔡田铺一期、塘西河一期、十五里河、职教城、科学城污水处理厂及清溪路垃圾填埋场综合治理工程全部竣工。完成道路绿化整治改造54条，实施大蜀山森林公园二期、逍遥津公园扩绿、三国遗址公园及蜀峰湾公园续建、城市景观水体污染治理等工程，新增园林绿地1。1万亩，新建、续建公园30个。44条道路景观整治及亮化工程同步实施。工业废水达标排放率进一步提高；大气污染防治取得新进展，集中拆除市区燃煤锅炉52台。公交线网进一步优化，水电气热等公用事业保障能力大幅提升。城市承载力及人居环境进一步改善。建筑业实现总产值突破800亿元，增长30%以上。

　　"141"组团联动推进。滨湖三校二期投入使用，滨湖世纪城等大型生态居住区相继开工，巢湖沿岸生态治理工程及路网等基础设施进一步完善，滨湖新区"城湖辉映"初步展现。政务文化新区功能加速拓展，八中新校顺利开学，大剧院及生活、商业等配套设施加快建设。老城区及城中村、危旧小区改造稳步实施，安纺一、三、四村及劳动村等改造工程即将完工。四大组团建设亮点纷呈，与主城区同步推进。

　　(四)夯实创新平台、完善政策体系，自主创新能力不断增强

　　根据合芜蚌自主创新综合配套改革试验区规划，结合我市科技创新试点市建设，制定实施方案，出台配套政策，设立专项资金，积极启动相关工作。争取省政府试验区建设资金2亿元，支持8个自主创新重大项目。

　　坚持突出企业创新，大力推进科技创新型企业培育计划，新增国家、省、市级创新型试点企业47家，新建省、市级工程技术研究中心15家，新增国家、省重点新产品33个。设立市创业风险投资引导基金，多渠道增加创新投入，重点支持一批成长型高新技术企业发展。充分发挥科技应用技术与开发资金引导作用，以电子信息、软件、新材料、节能环保等领域为重点，集中实施重大科技产业化项目31个。

　　加快科技创新载体与平台建设。建成五个科技创新公共服务中心子平台，促成产学研交易项目126个。围绕八大产业，以合力叉车等14家企业为核心，组建一批产学研战略联盟。试点市示范区总体规划基本完成，主干路网建设及项目落地取得重大进展。中国(合肥)自主创新要素对接会实现提档升级，签约项目62个、总额480。4亿元。专利授权量超过1000件，成为全国专利技术展示交易中心。

　　全年全社会研发投入占GDP比重达2%。新认定创新型企业45家、高新技术企业60家，高新技术产业产值突破1000亿元。

　　(五)推进体制机制创新，改革开放实现新跨越

　　推进新一轮思想大解放，不断深化各项重点改革。以合钢、国风、市属化工企业等12户大企业大集团为核心，国有、集体企业改革攻坚取得重大突破，208户企业实现转机建制，除公用事业企业外，市属国企改革基本结束，职工得到妥善安置。国有资产转让重组成效显著，建总行增资扩股兴泰信托，中盐总公司重组搬迁市属化工企业。国资监管力度明显加大，国有资本经营收益收缴、国有企业经营目标责任制、领导人员聘任制等制度顺利实施。非公经济快速发展，全年新增私营企业8500多家、个体工商户24000多户。在全国率先试行公务员跨部门交流轮岗，市直82个部门1632名公务员走上新岗位，交流轮岗面达到25。8%。建成全市企业基础信息共享和应用系统，建立部门联动机制，依法加强税收征管。深化部门预算改革，完善行政事业单位支出及资产管理，全面推进会计集中核算向国库集中支付转轨试点。设立市级偿债基金专户，支持县区融资担保。合肥城建、科大讯飞成功上市；试办村镇银行，新办小额贷款公司11家，新增典当企业9家，发行企业债券融资27亿元。完善管理机制，招投标市场实现交易额245亿元，节约和增值资金60亿元。成功完成取缔正三轮车非法营运工作，既保障了残疾人合法权益，又整治了城市交通秩序与环境。新一轮出租汽车经营权转换工作平稳实施，42家企业整合重组为5家，首批投放新增运力635台。改革城市管理体制，积极推进市政设施和道路绿化管养市场化。完善建设用地"双向约束"和工业项目用地快速反应机制，促进依法供地和节约集约用地。

　　坚持扩大开放。加强与长三角、珠三角等沿海发达地区及周边城市的交流合作，与智利圣地亚哥、韩国瑞山及温州缔结为友好城市。整合招商资源，提升招商实效，组建第五批招商小组537个，多层次、高密度开展境内外招商活动，实现招商引资782亿元，增长36。8%；新批外商投资企业69户，实际到位外资12亿美元，增长18。6%。高新、经开、新站三大开发区共引进资金近200亿元。鼓励企业"走出去"开拓国际市场，实现进出口总额75亿美元，增长20%。

　　(六)坚持民生优先，社会建设取得新进展

　　围绕争创全国文明城市，深入开展农贸市场、老旧小区、道路交通等专项整治行动，建立网格化城市管理体系，市容市貌显著改善。大力弘扬社会主义荣辱观，在全社会开展评选道德模范，树立20位道德楷模。深入推进学习型城市建设，开办庐州讲坛9期，引导开展全民读书活动。

　　实施28项民生工程，全年各级财政投入资金28。4亿元，惠及城乡居民400万人。实施"零就业家庭"援助工程，坚持创业促进就业，全年新增城镇就业人口8。9万人，超额完成1。9万人。农村低保实现提标扩面，保障人数超过10万人。实行被征地农民养老保障市级统筹，保障标准提高到每人每月260元，惠及18。2万人，基本实现应保尽保。城镇五项社会保险参保人数307。8万人次，新增60。8万人次；城镇居民基本医疗保障制度参保人数突破95万人；新型农村合作医疗制度参合率96%；18。3万人次享受城乡医疗救助。农村"515敬老工程"建设取得突破，新增床位超历年之和；县、区散居五保对象供养标准分别高于省标260元和300元。做好低收入阶层住房保障，新开工蓝天公寓等廉租房项目六个、总面积近9万平方米；建成经济适用房17万平方米。加强物价监管和调控，市场供应稳定有序；严厉打击制假售假行为，食品药品监管力度进一步加大。

　　继续推进优质教育资源均衡发展。免除53。5万中小学生义务教育阶段学费，外来务工子女定点学校增加到77所。市区新建、续建中小学24所，改造农村学校D类危房3。6万平方米。职教基地建设快速推进，17所院校签约入驻，完成投资10亿元。举办首届技工节，年培养各类技能人才15万人。合肥学院教科研水平有新提高，三联学院成功晋升本科层次。农村及城市社区卫生体系建设取得突破性进展，市中心医院及市二院、三院、合钢医院续建、扩建加快推进，改扩建乡镇卫生院、村卫生室194个。完善疾病预防控制和基层卫生监督网络，卫生应急处置能力全面提高。制定实施文化中长期发展规划。文艺创作精彩纷呈，涌现出庐剧《李清照》、《村长娘子》等一批优秀作品。数字电视整体转换工程突破34万用户，城乡广播电视综合覆盖率达100%。开展"全民健身与奥运同行"等主题活动，市九运会成果丰硕，第四届全国体育大会筹备工作扎实推进。人口和计划生育工作继续加强，国防教育和后备力量建设扎实推进，第七届村民委员会换届选举工作圆满完成。

　　完善治安管控体系，及时有效处置涉稳事件。出色完成奥运安保任务，强势开展严打整治，八类主要刑事案件下降21。8%。落实安全生产"两个主体"责任，积极开展"隐患治理年"活动，安全生产形势总体稳定。大力推进应急管理体系建设，组织各类应急演练800多次，政府应急能力明显提高。扎实开展矛盾纠纷排查化解、信访疑难复杂问题终结息访和重信重访问题专项治理工作，信访形势持续好转。在全国首创农民工普法学校，"五五"普法受到国家检查组充分肯定。民族宗教工作力度明显加大，在构建和谐合肥中的作用显著增强。

　　此外，外事、侨务、对台，以及妇女儿童、未成年人保护、老龄事业都有新发展，防震减灾、人防、消防、档案、地方志、保密、气象等工作也取得新成绩。

　　(七)坚持依法行政、效能优先，政府自身建设进一步加强

　　强力推进依法行政，坚持科学决策、民主决策，自觉接受人大的依法监督和政协的民主监督，全年共办理人大代表议案和建议152件、政协提案388件。加强同各民主党派、工商联、无党派人士和各人民团体的联系，主动听取和采纳各界人士的批评、建议和意见。完善《政府工作规则》，整合政府与部门法律顾问资源，加强政府类合同审查监督。完成地方性法规草案、政府规章各8件，清理政府规范性文件851件。依法办理行政复议案件65件，在部分单位试行行政首长出庭应诉制度。严格按照法定权限和程序行使权力，规范行政处罚自由裁量权。

　　继续深化效能建设，发展环境不断优化。85条政务和公用事业类服务热线统一整合为"12345"服务平台，实现了"一个号码找政府"的服务格局，在全国引起较好反响。改进窗口服务方式，推行热线受理首接负责制，全面实施并联办理制、全程代理代办制和柜员制。大力合并精简会议，减轻基层负担。严控出国组团，加强机关差旅费及后勤管理，进一步减少行政支出。推行"一岗双责"，改进政府新闻发布制度；完善政务公开工作机制，全市1306家单位发布了政府信息公开指南。加强电子政务建设，着力构建行政权力公开高效运行机制；完成"中国·合肥"门户网及60个部门子站改版，公共在线服务事项由106项扩充至近3000项；政务信息处理平台应用覆盖市、县，在全省率先实现"乡乡有网站"。深入开展反腐倡廉制度推进年活动，清理相关制度2859项。扎实推进政风行风评议，认真实施审计监督"双百"制度，加大查办案件工作力度。

　　各位代表，2008年，我们经受了一系列重大事件的考验。我们坚持以人为本、科学决策，千方百计保畅通、保供应、保民生、保稳定、保发展。汶川大地震发生后，我们坚决贯彻中央和省委省政府的决策部署，视灾情为命令，视援助为己任，全力开展对口救灾工作，先后派出5批368名干警、57名医务人员，圆满完成5222套过渡房和30所学校援建任务，收治灾区伤员34名，捐赠款物1。1亿元，为抗震救灾工作取得重大阶段性胜利作出了积极贡献，受到党中央、国务院的表彰，充分展示了合肥新形象和市民的新风貌。

　　回顾过去一年，成绩来之不易。这是党中央、国务院、省委、省政府和中共合肥市委正确领导的结果，是市人大依法监督、市政协民主监督、社会各界大力支持的结果，是全市人民同舟共济、奋力拼搏的结果。在此，我代表市人民政府，向全市广大工人、农民、知识分子和各级干部，向驻肥人民解放军指战员、武警官兵、公安干警和中央驻肥单位，向关心和支持政府工作的各民主党派、工商联、各人民团体和社会各界人士，表示衷心的感谢！

　　在看到成绩的同时，我们也清醒地认识到，与现代化滨湖大城市建设的要求相比，我们的工作还存在很大差距，制约合肥经济社会发展的一些长期性问题、深层次矛盾依然存在：经济规模不够大，农村经济基础比较薄弱，服务业发展尚显滞后，自主创新的潜能有待进一步释放；公共安全、城市管理等方面有待进一步加强；少数公务员思想作风和业务能力与新形势、新任务的要求还不相适应，等等。对此，我们将继续高度重视，采取切实措施加以逐步解决，永不自满、永不懈怠地做好各项工作，不辜负全市人民的期望和重托。

   二、2009年政府工作

　　2009年，是深入学习实践科学发展观、继续推进现代化滨湖大城市建设极其重要的一年，也将是新世纪以来我国经济发展最为困难的一年。由美国次贷危机所引发的国际金融危机，对世界乃至我国实体经济的影响正在加深，对我市经济发展影响的时间和程度存在很大的不确定性。受出口下降和消费信心不足影响，今年上半年经济运行中的一些困难和问题还难以预料，我们将面临着严峻的考验和挑战，保增长将是今年经济工作的首要任务。

　　2009年又将是合肥抢抓机遇、加快实现跨越的一年。一段时期以来，国家已采取一系列进一步扩大内需、促进经济增长的政策措施，全力保持经济平稳较快发展，果断实施积极的财政政策和适度宽松的货币政策，积极效应正在显现。尤其是要看到，经过改革开放30年的快速发展，特别是近几年的发展，一大批牵动性强的项目相继建成投产，城市发展的综合实力和竞争力大为增强、支撑条件和内在活力显著提升，合肥加快发展的基本面没有改变。只要我们坚定信心，积极应对，主动作为，趋利避害，就一定能战胜前进中的困难，保持经济平稳较快发展的势头。

　　2009年工作总体要求是：继续高举中国特色社会主义伟大旗帜，以邓小平理论和"三个代表"重要思想为指导，以深入开展学习实践科学发展观活动为动力，全面贯彻党的十七大、十七届三中全会、中央经济工作会议和胡锦涛总书记视察安徽重要讲话精神，按照省委八届九次全会和市委九届七次全会的部署要求，把保持经济平稳较快增长作为首要任务和头等大事。在工作推进中，我们要全力把强化政府服务作为保增长的组织保障，把加大各级财力投入作为保增长的政策支撑，把加强银政企合作作为保增长的重要手段，把突出为企业服务和项目推进作为保增长的着力点，把深化改革创新作为保增长的强大动力，把推动全民创业作为保增长的重要途径，把改善民生作为保增长的出发点和落脚点，努力确保全市经济社会持续稳定快速发展。

　　2009年全市经济社会发展的主要预期目标是：地区生产总值增长12%以上；财政收入增长13%；全社会固定资产投资增长21%以上；实际利用外资增长10%；社会消费品零售总额增长16%；城镇居民人均可支配收入增长10%；农民人均纯收入增长13%；城镇登记失业率控制在4。5%以内；人口出生率控制在12‰以内；居民消费价格指数涨幅低于2008年实际涨幅；单位GDP能耗及主要污染物减排量达到省控目标。

　　实现上述目标，今年我们将着重抓好以下九个方面工作：

　　(一)突出以项目为抓手，不断扩大有效投入

　　把狠抓项目、扩大投资作为保增长的主攻方向和着力点，紧紧抓住国家扩大内需、增加投资规模的机遇，围绕交通等重大基础设施、"三农"、技术进步、自主创新、节能减排和现代服务业等，谋划和筛选一批重大项目，最大限度地争取国家和省里的支持。继续推进省"861"及市"1346"行动计划重点项目建设。进一步完善项目调度机制，实施领导联系领衔负责、督查督办等制度。强化规划、土地、环境等综合配套服务，及时解决项目建设中存在的问题。

　　坚持扩大开放，积极参与泛长三角区域经济合作与分工，突出产业集群和产业链招商，不断拓宽招商新领域。继续推进"国家级汽车及零部件出口基地城市"、"服务外包基地城市"和"中部地区加工贸易梯度转移重点承接地"建设，积极申报设立出口加工区。进一步整合招商资源，市、县区形成合力，联动推进；全力办好第四届中博会和第五届徽商大会等重大会展活动，提升引资实效。加大企业上市辅导和扶持力度，推动优势企业上市融资。创造公平的市场环境，营造宽松的创新环境，激活和扩大民间投资。

　　(二)加快农村改革和发展，推进城乡一体化

　　围绕建设社会主义新农村，深化农村综合改革，加快城市产业向县域转移、基础设施向县域延伸、公共服务向农村覆盖，形成城乡一体化发展新格局。

　　着力在农村体制改革关键环节上取得突破。创新机制，加强土地承包经营权流转管理和服务，积极开展承包地换社保、宅基地换住房试点工作。完善农村宅基地制度，进一步推进征地制度改革。加大政策扶持力度，支持发展形式多样的农村合作经济组织和村镇银行、小额贷款公司等。继续推进乡镇政府职能转变，完善全程代理服务，提高社会管理和公共服务水平。深化农技推广体制改革，健全农业社会化服务体系。

　　坚持统筹城乡资源，大力支持三县经济做大做强。加快推进县域工业化，大力发展食品及农副产品加工业、配套产业和先进制造业，县域工业增加值达180亿元以上。深入推进合作共建，完善县区工业园区功能，着力培育一批特色园区，打造县域经济活力板块。科学编制现代农业发展规划，优化农业发展布局；加快发展特色种植业，新增设施栽培5000亩、露地菜一万亩；加快发展现代养殖业，新增规模养殖小区260个，养殖业占农业比重达到48%；加快发展林木花卉业，新增苗木花卉生产基地2。6万亩；加快发展休闲观光农业，重点建设牛角大圩、丰乐生态园等一批农业主题公园。

　　深入推进新农村建设。扩大"整村推进、连片整治"规模，重点实施10个万亩土地整理和新农村建设项目，加快缩并自然村、建设中心村。实施"文明乡村建设三年规划"，改善农村生产生活面貌。继续强化农村道路、水利、通讯、网络等基础设施建设，完成村村通水泥路，基本实现村村通班车，实施53座水库除险加固工程，解决农村17万人饮水安全问题。

　　(三)继续推进工业立市战略，加快构建现代工业产业体系

　　坚持一手抓新兴产业培育，一手抓传统产业改造，大力推动结构调整和产业升级，加快构建新型工业产业体系，着力建设先进的加工制造业基地和高新技术产业基地。

　　进一步加强与国内外大企业大集团的战略合作，积极争取企业总部调整在肥产业布局，扩展高附加值的产品系列；积极引进技术含量高、带动效应大的产业龙头项目及重大产业链延伸项目。全力推动京东方六代线、熔安动力、大陆轮胎、合肥中铝、马(合)公司冷轧薄板、合肥电厂6#机组、中盐化工、江汽两万辆客车等重大项目建设。集中力量发展电视平板显示器、发动机、汽车自动变速箱、冰箱和空调压缩机等关键配套产业，增强支柱产业的核心竞争能力。积极培育电子信息、新材料、新能源、节能环保、软件、公共安全等新兴产业，着力支持一批骨干企业提升在细分市场的规模优势和竞争力。大力发展循环经济，积极推行清洁生产，继续抓好工业、建筑业等领域重点产业、企业节能工作。

　　集中财力和人力，加强对企业支持与服务。创新银政企合作方式，鼓励金融企业创新，创造一切条件发挥企业保增长的主体作用。实行"一企一策"个性化服务，千方百计帮助企业排忧解难。认真落实发展非公经济的各项政策措施，改善发展环境，促进中小企业加快发展。继续加强工业运行调度监测，做好水电气、物流及企业用工、用地等保障服务，促进增产增收增效。

　　(四)发挥龙头作用，强力推进自主创新综合配套改革试验区建设

　　以规划为指导、政策为支撑，以体制机制创新为动力，以提高创新能力为核心，以培育创新型产业为目标，加快各项配套改革，在合芜蚌自主创新综合配套改革试验区建设中发挥龙头作用，使改革创新成为保增长的强大动力。

　　继续坚持企业创新为主体，整合各类创新扶持资金，改革和优化使用方式，重点支持高成长创新型企业和重大创新项目。积极探索多元化的投融资机制，加大初创型科技企业扶持力度，努力形成一批拥有自主知识产权、具有核心竞争力的关键技术、名牌企业和优势产业。全年新增高新技术企业100家、创新型企业50家，专利授权量1550件，高新技术产业产值增长30%以上。继续以构建产学研联盟为载体，突破行政隶属、区划及所有制限制，着力探索省市共建、院企互动、军民对接的产学研长效合作机制。强化科技创新公共服务中心的功能，提升资源积聚能力。依托科技创新试点市示范区，规划建设面向全省、辐射全国的科技成果转化交易平台、创新创业园区。筹备召开第二次部际协调小组会议，争取国家对试点市工作的更大支持。创新承办工作机制，着力把中国(合肥)自主创新要素对接会打造成为全国知名的科技展会。围绕建设"创新型人才高地"，继续实施"3820"产业英才建设等工程。提升知识产权创造、运用、保护和管理能力，加快重大专利技术产业化。

　　全方位推进体制机制改革创新。继续开展事业单位分类改革，建立健全机关公务员和事业单位聘用人员进出机制。加速国企改革重组步伐，着力优化国有经济结构，完善公司法人治理，建立健全现代企业制度。加强行政事业单位国有资产管理。强化依法治税，深化预算管理改革，推进公车管理制度改革，严格预算支出管理，严格控制一般性支出，减少一切不必要开支。进一步改革各专项资金使用方式，简化程序，阳光操作，整合财政资金，集中财力办大事，增强使用效益。清理经营性收费项目，合并、取消、降低一批收费项目和标准。合理划分市、区两级城市建设与管理事权，推进城市管理重心下移、属地管理，加快园林、市政等行业管办分离步伐。全面开展节约集约用地试点工作，改革现行土地申报与监管体制，完成新一轮土地利用总体规划修编，建立土地资源节约集约利用的新机制。

　　(五)继续开展大建设，打造生态宜居城市

　　在不断提升城市综合承载力的基础上，更加注重民生基础设施、区域综合交通枢纽和生态环境建设，完善城市综合服务功能。在更高层面上推动区域发展规划，加快省会经济圈和合淮同城化建设步伐。

　　突出以铁路为中心的综合交通枢纽建设。全面启动火车站改造、北货场新建工程，加快建设高铁站、高铁南环线、合蚌高铁、新桥国际机场、合肥新港及通江航道工程。继续加快城市出入口道路建设和改造步伐，完成南岗公路、合店路一期改造。

　　完善"141"组团路网联接，适时开工建设轨道交通一号线。进一步完善城区路网建设。开建东、西高架桥，续建畅通一环、二环改造工程，完善三环七射加方格路网骨架。中博会前完成24条道路景观整治亮化及一环沿线桥梁亮化工程。

　　继续以"四个优先"推进滨湖新区建设，加快建设总部经济区、金融服务综合区，推进渡江战役纪念馆、安徽名人馆、合肥博物馆、国际创新展示馆、城市规划馆等标志性馆群建设。积极建设政务文化新区文化艺术中心等一批高档次、综合型文化设施，加快提升生活、商业等配套服务水平，进一步集聚人气。

　　做好"水"、"绿"文章，强化生态城市建设。全面推动城市道路绿化提质改造，大力推进100公里外环森林生态绿色长廊二期工程建设；建成大蜀山森林公园、三国遗址公园和四里河、十五里河沿岸生态绿化工程；继续实施高压走廊绿化；全年新建园林绿地一万亩。完成西南部生态补水通道建设，实施董铺水库溢洪道扩建及南淝河生态补水等工程，加快塘西河二期及巢湖岸线生态整治，完成十五里河、二十埠河、板桥河上游截污工程，打造生态廊道和滨水景观。继续加快污水处理设施建设，力争完成蔡田铺一期续建、小仓房、经开区二期等五座污水处理厂及配套管网建设。加快开工污泥处置和生活垃圾焚烧发电项目，建立扬尘控制区，加大机动车尾气治理力度。

　　加快水电气热等公用事业发展，完成六水厂、南区热源二期等工程，尽快实现"川气东送"入肥。继续推进劳动村及一批拆迁恢复点建设，建成万和·新城、凤阳二村等14个危旧房改造项目。

　　(六)以现代服务业为突破口，大力提升城市综合竞争力

　　坚持从全局和战略的高度，把发展现代服务业作为深入实施"工业立市"战略的重要组成部分，推动服务业与制造业融合互动发展，延伸产业链，降低商务成本，提升城市综合竞争力。

　　大力发展生产性服务业。加快建设区域性金融中心，积极吸引国内外银行机构落户合肥，支持证券、保险、信托、基金管理公司等非银行金融机构发展。加强金融产品创新，鼓励企业发行债券，大力发展股权质押、互相担保、应收帐款质押等新型融资方式。加快建设区域性物流中心，以争创国家现代物流发展试点市为契机，提高服务水平，降低物流成本，重点扶持10个专业物流基地，支持物流企业通过重组转型、整合并购、战略联盟等方式做大做强。大力发展会展业，以举办中博会为契机，加快配套基础设施和服务体系建设，着力打造2-3个国内外知名会展品牌。积极承接服务业的国际转移，重点吸引服务业跨国公司在肥设立办事机构，引进现代服务项目和先进管理模式，带动服务业整体水平提高。加快完善城乡市场体系，继续抓好"万村千乡"和"新网工程"建设，全力推进"家电下乡"，全面提升为农服务水平。

　　大力发展面向民生的服务业。加快推进新老城区中央商务区及一批特色商业街区的建设和改造，支持建设一批超10万平方米的大型购物中心。推广连锁经营、特许经营等现代经营方式和新型业态。优化市场环境，支持和促进房地产市场健康稳定发展。编制旅游业发展总体规划，推进全省旅游中心城市建设。积极培育旅游市场主体，大力引进主题公园等现代大型旅游项目，突出抓好非物质文化遗产园等建设，打造三河等乡村文化旅游精品。壮大广播电视、新闻出版、文化娱乐、体育等产业，鼓励发展设计、策划等创意产业，大力推进动漫产业基地建设。建设一批社区服务中心，完善社区服务网络，推动社区服务产业化、社会化。

　　(七)优先实施民生工程，构建城乡社会保障体系

　　把民生工程摆在更加突出位置，继续增加财政投入，今年实施36项民生工程，努力让人民群众得到更多的实惠，共享改革发展的成果。

　　全力推进全民创业保增长促就业，进一步完善鼓励自然人创业相关支持政策体系。扩大技能培训规模，促进劳动者充分就业；建设返乡农民创业基地，争创全国创业型城市；完善就业援助制度，建立"零就业家庭"和困难群众再就业长效机制，全年新增城镇就业人员7万人。进一步加强城乡社会保障体系建设。加快建立新型农村养老保险制度，并与城镇职工养老保险有机对接。进一步完善城乡居民最低生活保障、被征地农民养老保障等制度。协调发展城镇职工基本医疗保险、城镇居民基本医疗保险、新型农村合作医疗和城乡医疗救助制度。完善劳动关系监管体系建设，切实维护农民工等群体合法权益。加快建立灾害应急救援和社会救助体系。积极实施老年公寓等惠民工程。推进"515敬老工程"建设，实现三县乡镇全覆盖。制定社区配套设施建设规划，全面建设和谐社区，争创"全国社区建设示范市"。开展新一轮廉租住房建设与租金补贴工作。

　　切实巩固教育优先发展地位，完善城乡教育经费保障机制。合理划分市、区教育管理权限，继续实施城区中小学布局优化调整，大力实施义务教育阶段学校标准化建设工程，落实困难家庭学生助学金和生源地助学贷款政策。大力推进职教基地建设，确保合肥幼师等市属职业学校一期工程建成交付使用。继续支持合肥学院建设和发展。深化医疗卫生体制改革，完善重大疾病防控网络，提高突发公共卫生事件应急处理能力。加强城市社区卫生服务网络和农村乡镇卫生院建设，改扩建村卫生室120个。加大文化艺术精品创作力度，广泛开展群众性文化活动。切实做好人口和计划生育工作，采取有力措施开展出生人口性别比综合治理。积极推动全民健身，促进市民身心健康。全面推进第四届全国体育大会筹备工作，加快建设轮滑场、室内球类馆和全民健身训练馆。完善国防动员体系，加强预备役部队和民兵建设，争创双拥模范城"七连冠"。创新民族宗教工作思路，打造全国杂散居地区民族工作样板市。完成全国第二次经济普查工作任务。做好外事、侨务、对台、人防、防震减灾工作，重视和推动档案、地方志、气象、红十字会、妇女儿童、未成年人保护、老龄和残疾人等事业发展。

　　(八)继续开展文明创建，深入推进和谐合肥建设

　　坚持用社会主义核心价值体系引领社会风尚，深入开展群众性精神文明创建活动。提升学习型城市建设水平，促进市民整体素质不断提高。加强城市管理，建立数字城市管理平台，建设智能交通系统；深入开展户外广告、"五小"行业、农贸市场整治行动，提升城市品位。开展文明交通、文明游园、文明上网、文明购物、文明施工等系列活动，着力提高细节文明水平。

　　继续深入开展平安创建活动。加强城乡社区警务工作，夯实基层警务基础。加强流动人口服务和管理，排查化解不安定因素，积极预防和妥善处置群体性事件。坚决依法打击各类违法犯罪活动，进一步增强人民群众的安全感。畅通信访渠道，完善党政领导干部信访接待日和信访听证制度，加强信访案件督办，建立联合接访新机制。加强县乡村三级信访工作网络建设，探索乡镇(街道)纪检、信访、综治、司法"四位一体"的信访工作新模式。继续深化法制宣传教育，推进"法治合肥"创建活动。

　　强化安全生产，严格安全责任追究制度，深入开展隐患排查治理，突出抓好道路交通、非煤矿山、危险化学品和烟花爆竹、建筑施工、人员密集场所等重点行业和领域的专项整治。继续开展产品质量和食品药品安全整治，力争所有的县级以上医疗机构、75%以上的乡镇卫生院、45%以上的村卫生室药房达到"规范药房"标准。进一步加强应急保障能力建设，构建社会应急网络。

　　(九)创新政府管理方式，提升服务发展能力

　　面对复杂多变的新形势和日益艰巨的新任务，全面完成今年经济社会发展的各项目标任务，必须进一步深化改革，创新政府管理体制和运行机制，不断把"效能革命"推向深入，努力提升服务科学发展的能力和水平。

　　在思想解放中提升能力。深入开展学习实践科学发展观活动，以思想的解放促进观念的转变、带动思路的创新、推动能力的提升。在思想解放过程中牢固树立科学发展观，围绕第一要务不动摇、不放松，强化大局意识和责任意识；用世界的眼光和战略的思维分析形势，提升准确把握形势、抢抓机遇的能力；以更宽的视野和创新的思维谋求发展，提升破解发展难题的能力；以敢想敢干、敢试敢闯的精神掌握发展主动权，提升服务发展的统筹协调能力；以能力的全面提升推动合肥的跨越式发展。

　　在深化改革中提升能力。深化行政管理体制改革，探索符合我市实际的、职能统一的大部门体制，理顺部门职责分工，健全部门间协调配合机制，确保政令畅通；继续开展行政审批制度改革，推进市行政服务中心"二次改革"，创新政府工作流程；按照科学化、规范化、法制化的要求，完善公务员交流轮岗配套制度；健全以行政首长为重点的行政问责制度，明确问责范围，规范问责程序，加大责任追究力度，提高政府公信力；优化公共资源配置，加强公共服务平台建设，着力营造良好的政务环境、市场环境、法制环境和人文环境。

　　在推进依法行政中提升能力。全面贯彻落实国务院《关于加强市县政府依法行政的决定》。加强制度建设，提高立法质量；强化行政执法责任制，细化量化行政裁量权；深化行政执法管理体制改革，拓宽相对集中处罚权的领域和范围；促进行政机关和工作人员严格按照法定权限和程序行使职权，履行职责。发挥专家咨询作用，广泛听取各方面的决策建议，科学民主决策。加强行政监督，自觉接受人大及其常委会的依法监督、政协民主监督，认真办理人大代表议案、建议和政协提案。主动听取并采纳各民主党派、工商联、无党派人士和各人民团体的意见，接受新闻媒体和社会公众监督。贯彻落实《政府信息公开条例》，继续深化政务公开。强化反腐倡廉制度建设，健全行政立法、决策、执行等环节的制度体系，形成以制度管人管事的长效机制。严防权力滥用，确保政务规范、政事清明、政风端正。

　　在狠抓落实中提升能力。进一步转变工作作风，深入基层、深入群众、深入实践；破除繁文缛节，精简事务活动；集中精力抓落实，凝心聚力保增长，心无旁骛促发展。坚决减少一般性会议和礼仪性活动，大力压缩会议时间和规模。对推进科学发展的各项举措，细化目标任务，明确工作措施，切实提高抓落实的效率和质量。以创新的精神促进落实，以实干的行动推进落实，把精力集中在想干事上，把本领体现在会干事上，把目标锁定在干成事上，使抓落实成为各级各部门的自觉行动。深入推进机关效能建设，建立健全督查到位、考核到人、一抓到底的工作落实机制，强化工作执行力和落实力。

　　各位代表，人民的信任和重托，是我们做好工作的强大动力。新的一年，孕育着新的希望。让我们紧密团结在以胡锦涛同志为总书记的党中央周围，高举中国特色社会主义伟大旗帜，全面贯彻落实科学发展观，在省委、省政府和中共合肥市委的坚强领导下，坚定信心，把握机遇，团结奋斗，扎实工作，为合肥的跨越式发展和现代化滨湖大城市建设而努力奋斗，以优异的成绩向国庆60周年献礼！
